# Machine Learning Integration Framework Constructs a Lactylation‐Associated Gene Signature to Improve Prognosis in Bladder Cancer

**DOI:** 10.1002/cam4.71477

**Published:** 2026-01-08

**Authors:** Jingsong Wang, Qianxue Lu, Panpan Jiao, Jun Jian, Qingyuan Zheng, Zhiyuan Chen, Xiuheng Liu, Shanshan Wan, Lei Wang

**Affiliations:** ^1^ Department of Urology Renmin Hospital of Wuhan University Wuhan Hubei China; ^2^ Institute of Urologic Disease Renmin Hospital of Wuhan University Wuhan China; ^3^ Department of Ophthalmology Renmin Hospital of Wuhan University Wuhan Hubei China

**Keywords:** bladder cancer, lactylation, machine learning integration, prognosis

## Abstract

**Background:**

Bladder cancer remains a significant challenge in oncology owing to its high recurrence rates and limited treatment options, particularly in cases of resistance to standard therapies.

**Aims:**

Our study aimed to pinpoint a lactylation‐associated gene signature capable of predicting prognosis and providing important theoretical support for drug development and precision therapy in bladder cancer patients.

**Materials and Methods:**

Leveraging RNA sequencing data from the TCGA and GEO databases, we scrutinized the expression profiles of lactylation‐associated genes and pinpointed a signature comprising eight genes strongly linked to prognosis based on a machine learning integrative framework. Our prognostic model, incorporating the expression levels of these lactylation‐associated genes, demonstrated high accuracy in predicting patient outcomes, including survival rates and response to immunotherapy. Furthermore, functional analyses revealed the potential mechanisms through which lactylation‐associated genes contribute to bladder cancer progression and treatment resistance. Further validation of the close association of these eight genes with bladder cancer was also confirmed through in vitro RT‐PCR experiments and Human Protein Atlas (HPA). The drug enrichment analysis and molecular docking provide us with potential drugs and their binding modes with target proteins. To further investigate the relationship between the model gene and bladder cancer, we conducted a series of in vitro experiments.

**Results:**

We found that knockdown of AHNAK reduced the proliferation, migration, and invasion abilities of bladder cancer cells and also promoted cell apoptosis.

**Discussion:**

Overall, our study highlights the importance of lactylation‐associated genes as prognostic markers and potential therapeutic targets in bladder cancer.

**Conclusion:**

Our identification of this gene signature lays the groundwork for personalized treatment strategies and enhanced patient management in clinical practice.

## 1 Introduction

Bladder cancer (BCa) is a prevalent malignancy globally, with high incidence and mortality rates [1]. The latest survey indicates that there are approximately 573,000 new cases of bladder malignancy and 213,000 deaths worldwide each year [2]. Existing research has not clearly identified the causative factors and specific pathogenic mechanisms of bladder cancer. It is known that smoking is a common risk factor for BCa, and occupational exposure to carcinogenic chemicals can also increase the risk of developing BCa [3, 4]. BCa is often categorized into non‐muscle‐invasive bladder cancer (NMIBC) and muscle‐invasive bladder cancer (MIBC) according to the depth of tumor invasion into the muscle tissue, with approximately 70% of new cases diagnosed as NMIBC [5, 6]. Currently, traditional transurethral resection of bladder tumor along with intravesical therapy has shown satisfactory therapeutic effects for NMIBC [7]. However, many patients experience recurrence or progression, resulting in poor treatment sensitivity and high mortality rates [8, 9]. The standard approach for MIBC is radical cystectomy or neoadjuvant chemotherapy followed by surgery. However, these approaches benefit only a subset of patients, and the highly invasive nature of the surgery will reduce the quality of life [10, 11]. Therefore, there is an urgent need to develop new biomarkers and treatment targets to improve BCa prognosis and prevent its recurrence and progression.

Protein lactylation, firstly reported by Zhang [12] and his team in 2019, represents a novel form of protein post‐translational modification. This modification involves attaching lactate molecules to lysine residues on proteins. The specific mechanism of lactylation remains an area of active research, but it is known to impact protein structure, stability, and function, thereby influencing various cellular processes [13]. Before this discovery, lactate was traditionally considered only as a metabolic waste product generated during glycolysis. Meanwhile, glycolysis is considered a momentous characteristic of energy metabolism in tumor cells, leading to lactate accumulation in the tumor microenvironment, which is defined as the “Warburg Effect” [14, 15]. Lactate accumulation alters the pH balance in the tumor vicinity and impacts the growth, invasion, and metastasis of tumor cells. Additionally, the Warburg effect reduces the dependency of tumor cells on oxygen, posing challenges for cancer treatment [16, 17]. Lactylation is believed to contribute significantly to the initiation and progression of tumors, jointly influencing the metabolic characteristics and biological behavior of tumor cells with the Warburg effect. Histone lactylation serves as the key mechanism by which lactate fulfills its functions and participates in critical biological processes [18]. Lactate was proved to promote tumorigenesis by regulating MOESIN lactylation and enhancing TGF‐β signaling in regulatory T cells. Thus, combination therapy with lactate dehydrogenase inhibitors may exhibit stronger anti‐tumor effects compared to using anti‐PD‐1 alone [19]. In hepatocellular carcinoma, demethylzeylasteral could inhibit tumorigenicity by restraining histone lactylation [20]. But there are relatively few reports on the relationship between lactylation and BCa. Xie et al. [21] found that CircXRN2 could constrain histone lactylation by stimulating the Hippo signal pathway to inhibit the progression of BCa. Additionally, lactylation has been linked to resistance mechanisms against platinum‐based chemotherapy in BCa. Further exploration of its role in BCa could uncover valuable insights for targeted therapies and treatment strategies [22].

Herein, our study constructed a prognostic signature based on lactylation‐associated genes, thereby exploring the correlation between lactylation‐associated genes and the prognosis of BCa patients as well as the tumor microenvironment. Our model could not only accurately assess the survival status of BCa patients independently but also predict their sensitivity to chemotherapy and immunotherapy drugs. Drug enrichment analysis identified potential candidate drugs that may affect specific disease processes by exploring interactions between genes or proteins. The final in vitro experiments further confirmed the feasibility of our model.

## 2 Materials and Methods

### 2.1 Data Collection and Organization

We downloaded mRNA matrix data, clinical data, and gene mutation annotation data of BCa patients from The Cancer Genome Atlas (TCGA) database (https://tcga‐data.nci.nih.gov/tcga/). Additionally, the datasets GSE13507 and GSE32894 from the Gene Expression Omnibus (GEO) database (https://www.ncbi.nlm.nih.gov/geo) were used for external validation. Samples with unclear survival status and incomplete clinical data from both datasets were excluded, leaving the remaining samples for further analysis. Based on existing reports, we obtained 332 lactylation‐related genes [23].

### 2.2 Identification of Differentially Expressed and Prognostic Genes

Using “limma” R package, we extracted mRNA data from TCGA database and identified differentially expressed lactylation‐related genes, with |logFC| > 1 and *p* < 0.05. Based on the clinical data, we performed univariate Cox (uni‐Cox) regression analysis to obtain lactylation‐related genes associated with overall survival in BCa patients. The standard for uni‐Cox analysis was set at *p* < 0.05.

### 2.3 Construction and Validation of Prognosis Risk Signature Based on Lactylation‐Related Genes

Subsequently, a lactylation‐related signature was developed using 10 machine learning algorithms and 101 algorithm combinations. The algorithms included Random Survival Forest (RSF), Elastic Net (Enet), Lasso, Ridge, Stepwise Cox, CoxBoost, Cox Partial Least Squares Regression (plsRcox), Supervised Principal Components (SuperPC), Generalized Boosted Regression Model (GBM), and Survival Support Vector Machine (survival‐SVM). Our machine learning algorithms followed the methodology reported in a previous study. The TCGA cohort was randomly divided into a test group and a validation group while two GEO cohorts were utilized as external validation cohorts. Harrell's concordance index (C‐index) was calculated across all cohorts. The machine learning combination model with the highest average C‐index score was deemed our optimal model. Then we calculated the risk score of each patient using the following formula:

$$\text{Risk score}=\sum_{i=1}^{n}\text{coefficient}\;{\text{of gene}}_{i}\times \text{expression}\;{\text{of gene}}_{i}.$$

All patients were categorized into low‐risk and high‐risk group according to their median risk scores. Kaplan–Meier analysis was utilized to measure the overall survival of BCa patients in two risk groups and the area under the receiver operating characteristic (ROC) and the concordance index were employed to assess the correctness of the risk signature for predicting prognosis. Terminally, we used uni‐ and multi‐Cox regression analyses to evaluate the independence of the risk signature from other clinical features.

### 2.4 Gene Functional Annotation Analysis

Using the “clusterProfiler” and “enrichplot” R packages, Gene Ontology (GO), Kyoto Encyclopedia of Genes and Genomes (KEGG), and Gene Set Enrichment Analysis (GSEA) analysis were performed to examine the functions and pathways enriched in differentially expressed lactylation‐related genes, with *p* < 0.05 and FDR < 0.25.

### 2.5 Immune Microenvironment and Tumor Mutation Burden (TMB)

To assess the tumor immune microenvironment, we utilized the CIBERSORT algorithm in two risk groups. We assessed the fraction of immunocyte infiltration and scored the immunocyte infiltration using the “limma”, “parallel”, “reshape2” and “ggpubr” R packages. Then the Single‐Sample GSEA (ssGSEA) and the “GSVA” R package were used to analyze the differences of immune function in two risk groups. Finally, we compared the expression level of several immune checkpoints in high‐ and low‐risk groups. The TMB of BCa patients in two risk groups was calculated based on somatic mutation data obtained from the TCGA database, and we compared the survival rate of high‐TMB and low‐TMB patients using the “survival” and “survminer” R packages.

### 2.6 Cluster Analysis and Drug Sensitivity Analysis

For cluster analysis and drug sensitivity analysis, we divided all BCa patients into two clusters using the “ConsensusClusterPlus” R package. We then compared survival status and the expression of various immune checkpoints between these clusters. Additionally, we determined the half‐maximal inhibitory concentration (IC50) of various chemotherapy and targeted drugs using the “oncoPredict” R package.

### 2.7 Drug Enrichment Analysis and Molecular Docking

We acquired the gene‐drug interaction datasets from the Drug Signatures Database (https://dsigdb.tanlab.org/). Using “limma” and “clusterProfiler” R packages, we identified therapeutic drugs associated with the prognostic genes. To further validate the interactions between the drugs and potential targets, we conducted molecular docking analysis. We downloaded the 3D structures of the proteins and drugs from the PubChem database (https://pubchem.ncbi.nlm.nih.gov/) and the Protein Data Bank (https://www.rcsb.org/), respectively. Docking simulations between the identified drugs and the target protein were performed using cavity‐detection guided Blind Docking software (https://cadd.labshare.cn/cb‐dock2/index.php).

### 2.8 Cell Culture and Treatment

The cell lines were all procured from the American‐Type Culture Collection (ATCC). The T24 and 5637 bladder cancer cell lines were cultured in RPMI 1640 supplemented with 10% fetal bovine serum media, while the human immortalized uroepithelial (SV‐HUC‐1) cell line was cultured in Ham's F‐12K/10% fetal bovine serum media. All cell lines were maintained in a 5% CO_2_ incubator at 37°C. Small interfering RNA (siRNA) was used to silence AHNAK in bladder cancer cells. Cells were seeded at a density of 2 × 10^5^ cells per well in 6‐well plates and incubated for 24 h until they reached 60%–70% confluence. Lipofectamine 6000 (Beyotime, Shanghai, China) transfection reagent was used according to the manufacturer's instructions. After 24–48 h of incubation, total RNA was extracted using TRIzol reagent and gene expression levels were assessed by RT‐qPCR.

### 2.9 RNA Extraction and RT‐qPCR

The total RNA was extracted from the three cell lines using TRIzol reagent and reverse‐transcribed into cDNA using a reverse transcription kit from Servicebio. RT‐qPCR was then performed on the Quanta gene q225 real‐time PCR system. The primer sequences used are listed in Table 1.

**TABLE 1:** Basic information about all primers used in RT‐PCR experiments.

Primer information	Primers	Primer sequence (5′‐3′)
NM_001346445.2	H‐AHNAK‐S	AGTGGTTCTGAGCGGGGAT
	H‐AHNAK‐A	AATTCAGGGCTACTGATGTCTATGT
NM_001363669.2	H‐CALM1‐S	TGAAGTGGATGCTGATGGTAATG
	H‐CALM1‐A	GTCAAAGACTCGGAATGCCTCA
NM_004343.4	H‐CALR‐S	CCTTGGAAGACGATTGGGACT
	H‐CALR‐A	CGCCAAAGTTATCATAGGCATAGA
NM_000402.4	H‐G6PD‐S	CACATCTCCTCCCTGTTCCGT
	H‐G6PD‐A	CTGTTGGCAAATCTCAGCACC
NM_004342.7	H‐CALM1‐S	CTGTTCCTGCTGAAGGTGTACG
	H‐CALM1‐A	CCTACCTTCAAGCCAGCAGTTTC
NM_003380.5	H‐vimentin(1)‐S	ATCTGGATTCACTCCCTCTGGTT
VIM	H‐vimentin(1)‐A	CGTGATGCTGAGAAGTTTCGTTG
NM_002046	H‐GAPDH‐S	GGAAGCTTGTCATCAATGGAAATC
	H‐GAPDH‐A	TGATGACCCTTTTGGCTCCC
NM_0025862.3	H‐CACYBP‐S	CTCCCATTACAACGGGCTATAC
	H‐CACYBP‐A	GAACTGCCTTCCACAGAGATG
NM_000507.4	H‐SATB1‐S	GATCATTTGAACGAGGCAACTCA
	H‐SATB1‐A	TGGACCCTTCGGATCACTCA

### 2.10 Wound Healing Assay

Cells were seeded in 6‐well culture plates and allowed to reach near confluence (approximately 100%) under standard growth conditions. A sterile pipette tip was carefully employed to induce a linear scratch across the cell monolayer, creating a controlled wound. Following the injury, the monolayer was gently washed with phosphate‐buffered saline (PBS) to remove any cellular debris and subsequently cultured in serum‐free medium to inhibit cell proliferation and facilitate the study of migratory behavior. Wound healing was monitored at two distinct time points: immediately after wounding (0 h) and after 24 h of incubation. Phase‐contrast images of the wound area were captured at each time point using a light microscope to evaluate the rate of wound closure and the migratory response of the cells.

### 2.11 EdU (5‐Ethynyl‐2′‐Deoxyuridine) Assay

Cell proliferation was measured using the EdU (5‐Ethynyl‐2′‐deoxyuridine) assay. Cells were seeded and cultured until reaching the desired confluence. EdU was added to the culture medium at a final concentration of 10 μM and incubated for 2 h to allow incorporation during DNA synthesis. After incubation, cells were fixed with 4% paraformaldehyde and permeabilized with 0.5% Triton X‐100. EdU incorporation was detected using a fluorescent azide via click chemistry, and nuclei were counterstained with Hoechst. Proliferating cells were identified by their EdU‐positive staining, and the percentage of proliferating cells was calculated by counting EdU‐positive cells relative to total Hoechst‐stained cells.

### 2.12 Transwell Invasion Assay

Cell invasion was evaluated using Matrigel‐coated Transwell inserts with 8‐μm pores (Corning, USA). A suspension of 1 × 10^5^ cells in serum‐free medium was plated in the upper chamber, while the lower chamber was filled with medium supplemented with 10% FBS to act as a chemoattractant. After a 24‐h incubation at 37°C, non‐invading cells on the upper surface of the membrane were gently removed using a cotton swab. Cells that had invaded through the Matrigel matrix and membrane were fixed in 4% paraformaldehyde, stained with 0.1% crystal violet, and the number of invasive cells was quantified by counting in five random fields under a light microscope.

### 2.13 Apoptosis Assay

Cell apoptosis was detected using the Annexin V‐FITC/PI Apoptosis Detection Kit (Beyotime, China) according to the manufacturer's instructions. Briefly, cells were collected, washed twice with cold PBS, and resuspended in 195 μL of binding buffer. Then, 5 μL of Annexin V‐FITC and 10 μL of propidium iodide (PI) were added, followed by incubation at room temperature for 15 min in the dark. The stained cells were analyzed by flow cytometry, and the percentages of apoptotic cells were quantified using FlowJo software.

### 2.14 Statistics Analysis

All data was analyzed by R software (version 4.4.0) and GraphPad Prism 8.0. The Wilcox rank‐sum test was used to compare the differences between two groups. Statistical significance was determined as *p* < 0.05 for all two‐tailed tests (*p < 0.05, **p < 0.01, ***p < 0.001).

## 3 Results

### 3.1 Identification of Prognostic Lactylation‐Related Genes in BCa

**FIGURE 1:**
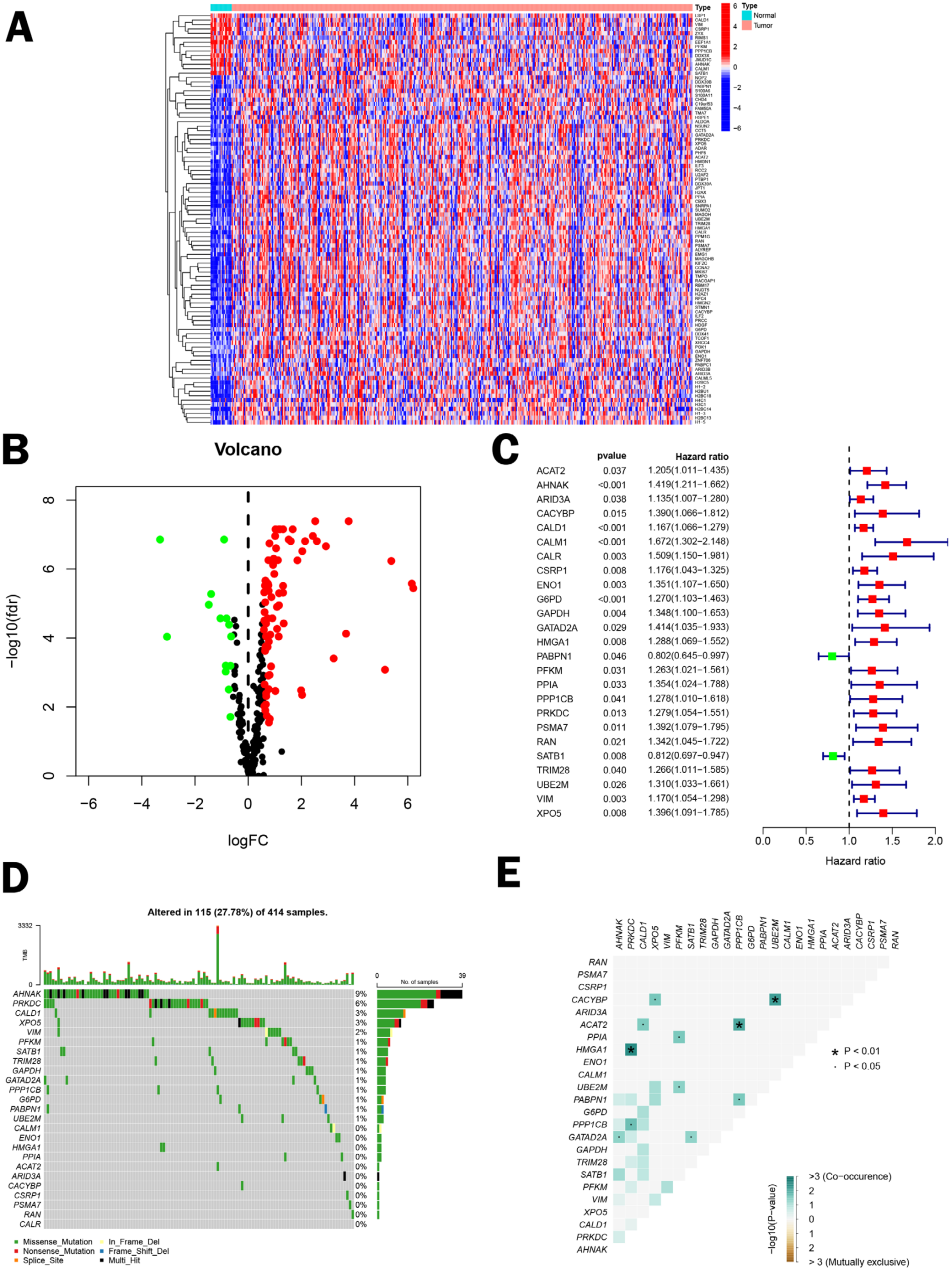
Identification of lactylation‐related genes. (A) Heatmap of differentially expressed lactylation‐related genes; (B) Volcano plot of differentially expressed lactylation‐related genes; (C) The forest plot of prognostic lactylation‐related genes based on uni‐Cox analysis; (D) Waterfall plot of somatic mutations in prognostic lactylation‐related genes; (E) Common mutations correlation among prognostic lactylation‐related genes.

The flowchart of our study was displayed in Figure S1. Firstly, we analyzed RNA‐seq data from TCGA‐BLCA, comprising 19 normal and 414 tumor samples. Differentially expressed lactylation‐related genes in BCa patients were visualized in Figure 1A, with a volcano plot of DEGs in Figure 1B. Next, utilizing uni‐Cox regression analysis, we identified 25 lactylation‐related genes associated with prognosis in BCa patients (Figure 1C). Then we investigated the frequency of somatic mutations in these 25 prognostic lactylation‐related genes, noting that 115 of 414 samples (27.78%) had mutations, with AHNAK having the highest frequency of mutation (Figure 1D). Meanwhile, Figure 1E illustrated the common mutations relationship among these genes.

### 3.2 Construction of a Lactylation‐Related Genes Prognostic Risk Signature

**FIGURE 2:**
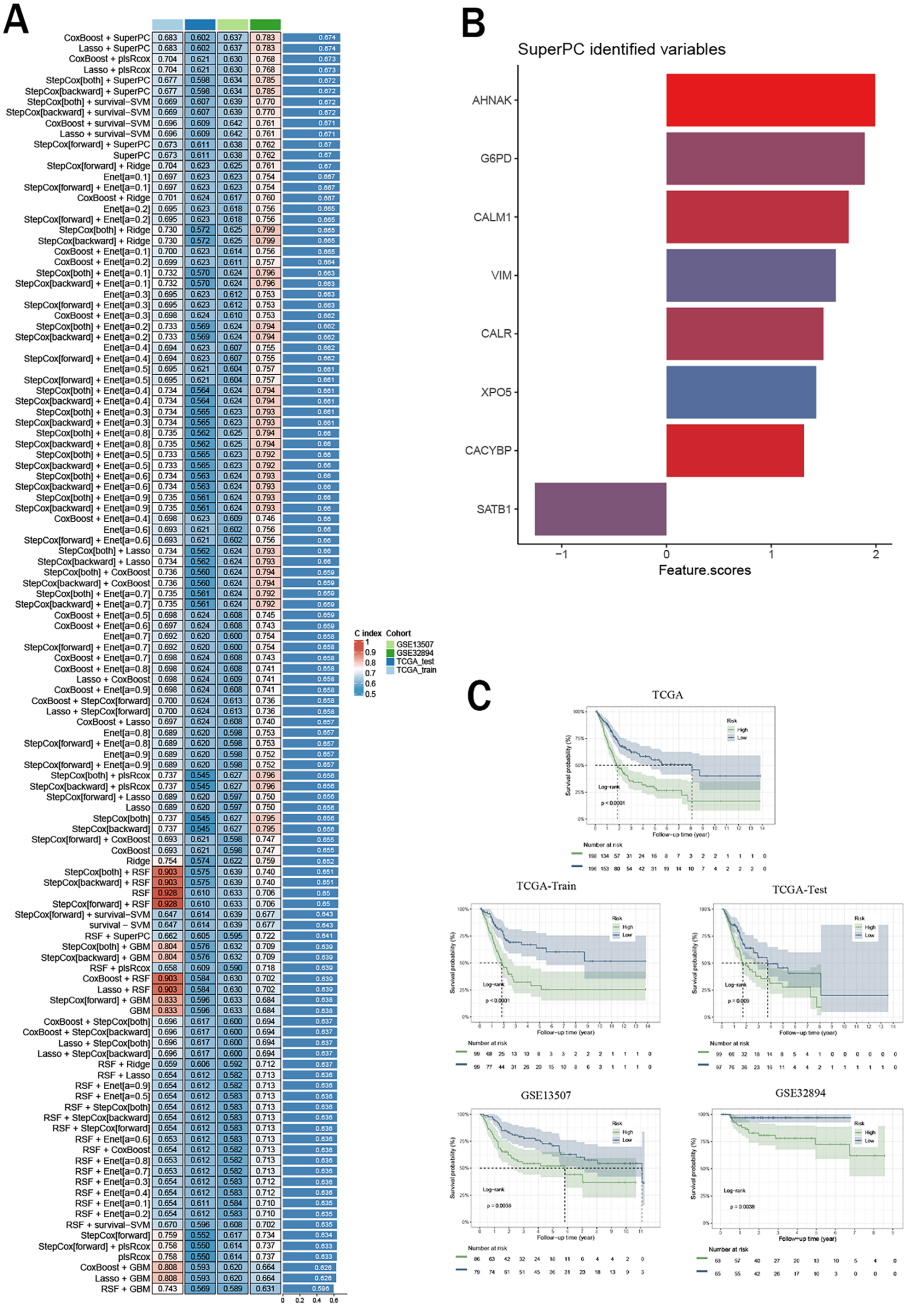
Construction of the prognostic risk signature. (A) The C‐index values of 101 machine learning combinations across all datasets; (B) The genes and their regression coefficients for the optimal model; (C) Survival analysis curves for all cohorts.

In the TCGA cohort, we fitted 101 machine learning combinations of predictive models using a leave‐one‐out cross‐validation framework and calculated the C‐index for each combination across all TCGA and GEO cohorts. We found that the combination of the CoxBoost and SuperPC methods resulted in the highest average C‐index across all cohorts. Ultimately, we constructed the optimal prognostic model using 8 genes (Figure 2A,B). Then we calculated the risk score for each BCa patient utilizing the following formula:

$$\begin{array}{c} \text{Risk}\;\text{score}=\left(\text{AHNAK}\times 0.219395117274817\right)+\left(\text{CALM}\times 0.0846915536656999\right)\\ \;+\left(\text{CALR}\times 0.0299443742371303\right)+\left(G6\mathrm{PD}\times 0.171443752349604\right)\\ +\left(\text{SATB}1\times -0.0623592468740203\right)+\left(\mathrm{VIM}\times 0.107602408181455\right)\\ +\left(\text{CACYBP}\times 0.109498539180355\right)+\left(\mathrm{XPO}5\times 0.0534965043102488\right). \end{array}$$

Based on these scores, all patients in the TCGA and GEO cohorts were categorized into high‐risk and low‐risk groups. Notably, patients in the low‐risk group exhibited better survival rates than those in the high‐risk group in all cohorts (Figure 2C).

### 3.3 Assessment of the Prognostic Risk Signature

**FIGURE 3:**
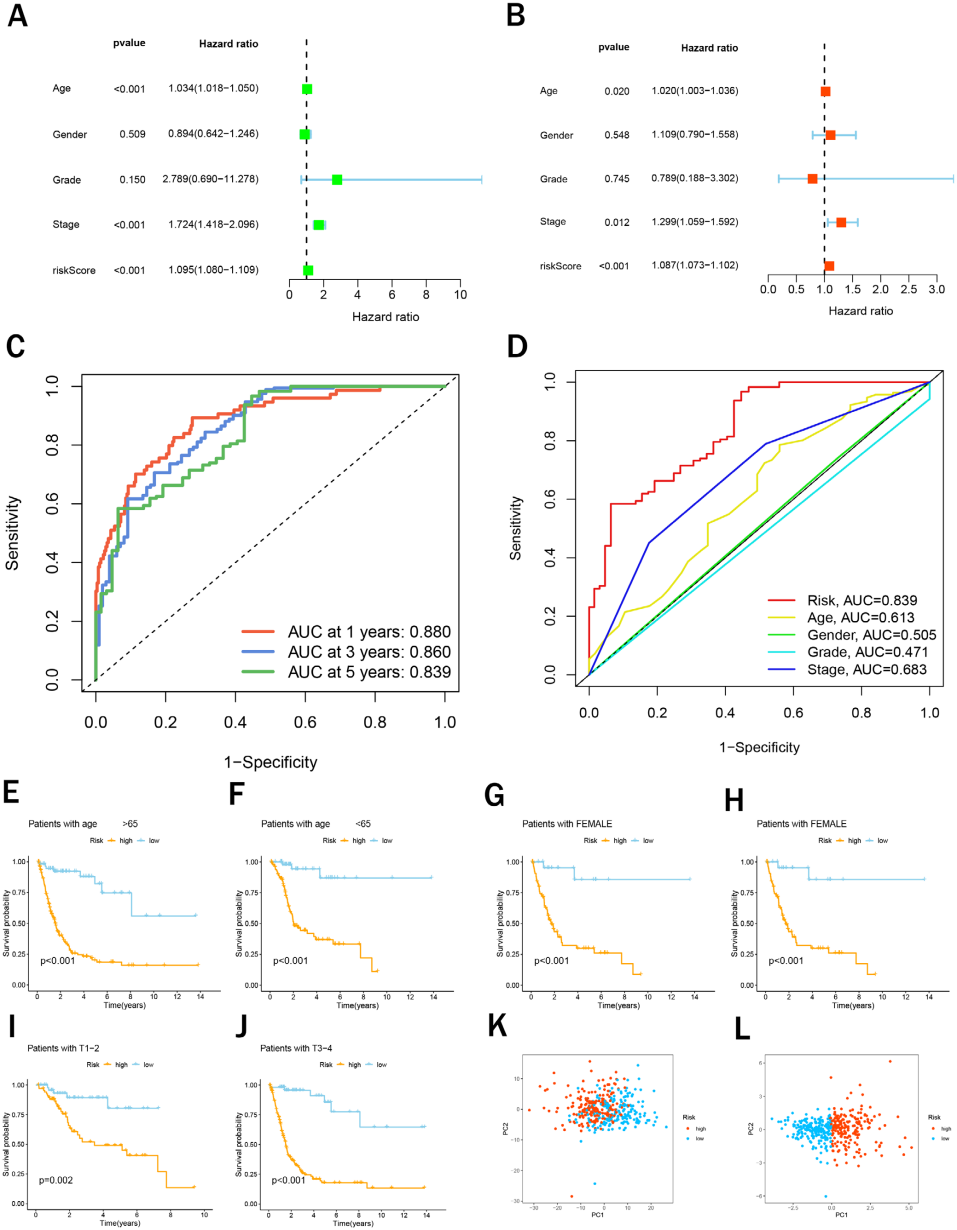
Validation of the risk signature. (A and B) Uni‐Cox and multi‐Cox regression analysis of clinical features and risk score in the TCGA cohort; (C) The receiver operating characteristic (ROC) curves of 1‐, 3‐, and 5‐year survival rates; (D) The ROC curves of the risk signature and clinical features. (E and F) The Kaplan–Meier (KM) survival analysis of patients with Age (≥ 65 and < 65) based on the risk signature; (G and H) The Kaplan–Meier (KM) survival analysis of patients with Gender (Female and Male) based on the risk signature; (I and J) The Kaplan–Meier (KM) survival analysis of patients with Stage (Stage 1–2 and Stage 3–4) based on the risk signature. (K) PCA of total lactylation‐related genes; (L) PCA of lactylation‐related genes used in the risk signature.

Uni‐ and multi‐Cox regression analyses were employed to assess the independent prognostic value and clinical correlations of our risk signature. The results suggested that risk score, age, and tumor clinical stage were all independent prognostic indicators (Figure 3A,B). Furthermore, we used the ROC curve analysis to further evaluate the predictive accuracy of our risk signature. The area under curves (AUCs) of the 1‐,3‐,5‐year survival rates were 0.880, 0.860, and 0.839 (Figure 3C), suggesting that the risk signature possessed a good prognostic predictive value in BCa patients. Importantly, the risk score's AUC (0.839) surpassed those of tumor stage (0.683), age (0.613), gender (0.505), and tumor grade (0.471), highlighting the superior prognostic predictability of our risk signature (Figure 3D). Then we performed a survival analysis in two risk groups based on the clinical features and found that patients in high‐risk groups exhibited a obviously poorer OS than those in low‐risk groups according to age (≥ 65 or < 65), gender (male or female), and clinical stages (stage I–II or stage III/IV), further confirming the independence of our risk signature in predicting BCa prognosis (Figure 3E–J). What's more, principal component analysis results indicated that our risk signature could effectively distinguish high‐risk and low‐risk BCa patients (Figure 3K,L).

### 3.4 Biological Functional Analysis

**FIGURE 4:**
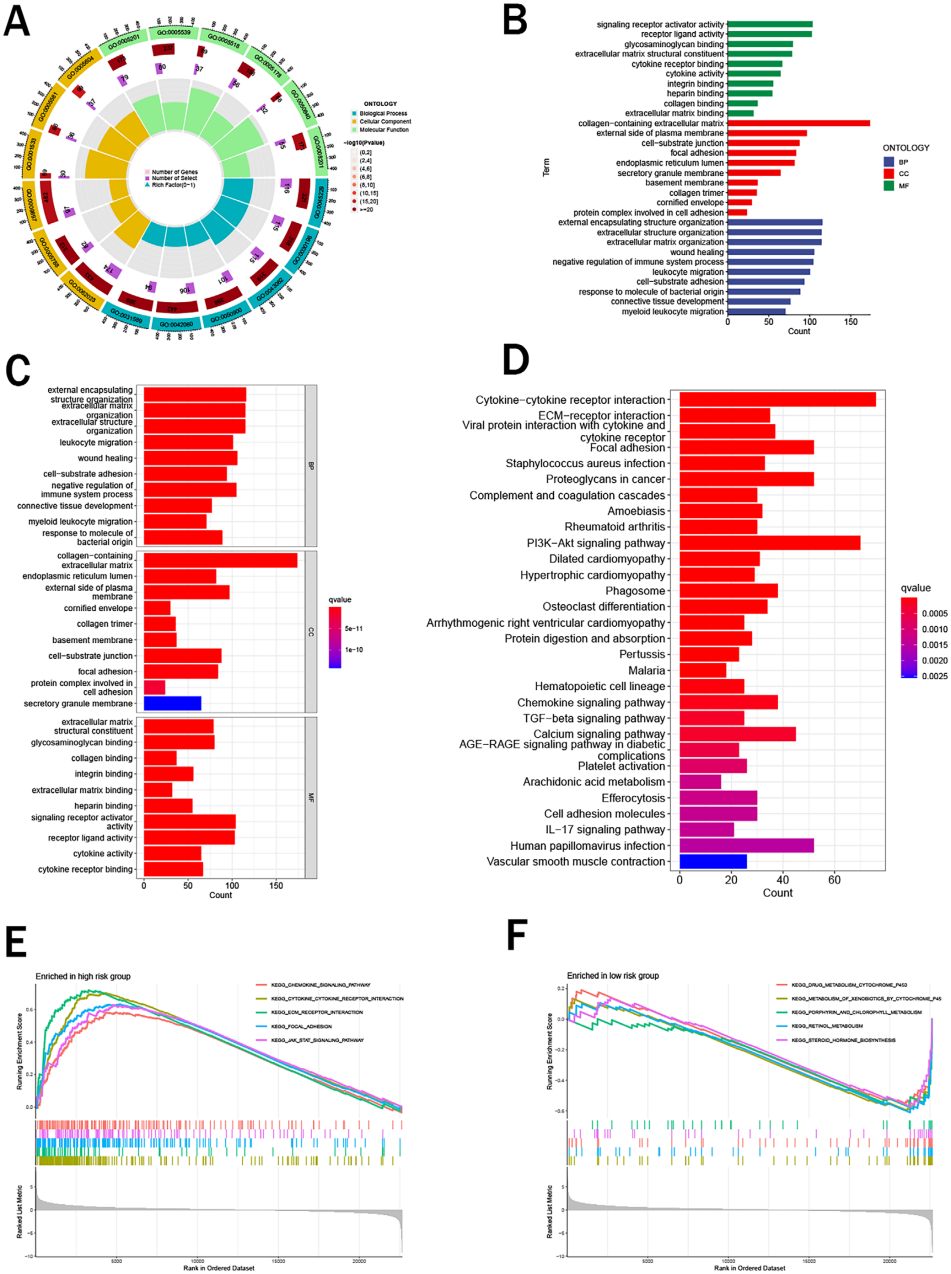
Biological functional and pathway enrichment analysis. (A–C) GO functional enrichment analysis; (D) KEGG pathways enrichment analysis; (E and F) GESA pathways enrichment analysis in high‐ and low‐ risk groups.

To explore the biological function of lactylation‐related genes, we performed GO, KEGG, and GSEA based on the risk signature. Our findings revealed enrichments in various biological processes (BP) associated with lactylation‐related genes, including external encapsulating structure organization, negative regulation of immune system process, leukocyte migration, and so on. The results displayed that various cellular components (CC), such as collagen−containing extracellular matrix, external side of plasma membrane, and cell−substrate junction, were much more abundant. In regard to molecular function (MF), signaling receptor activator activity, receptor ligand activity, and glycosaminoglycan binding exhibited the most enrichment (Figure 4A–C). The outcomes of KEGG clarified that many signaling pathways, including cytokine−cytokine receptor interaction, PI3K − Akt signaling pathway, proteoglycans in cancer, and ECM − receptor interaction, were closely associated with lactylation‐related genes, further elucidating their potential biological significance (Figure 4D). As shown in Figure 4E,F, the results of GSEA indicated several biological activity pathways were obviously enriched in two risk groups.

### 3.5 Tumor Immune Microenvironment and TMB

**FIGURE 5:**
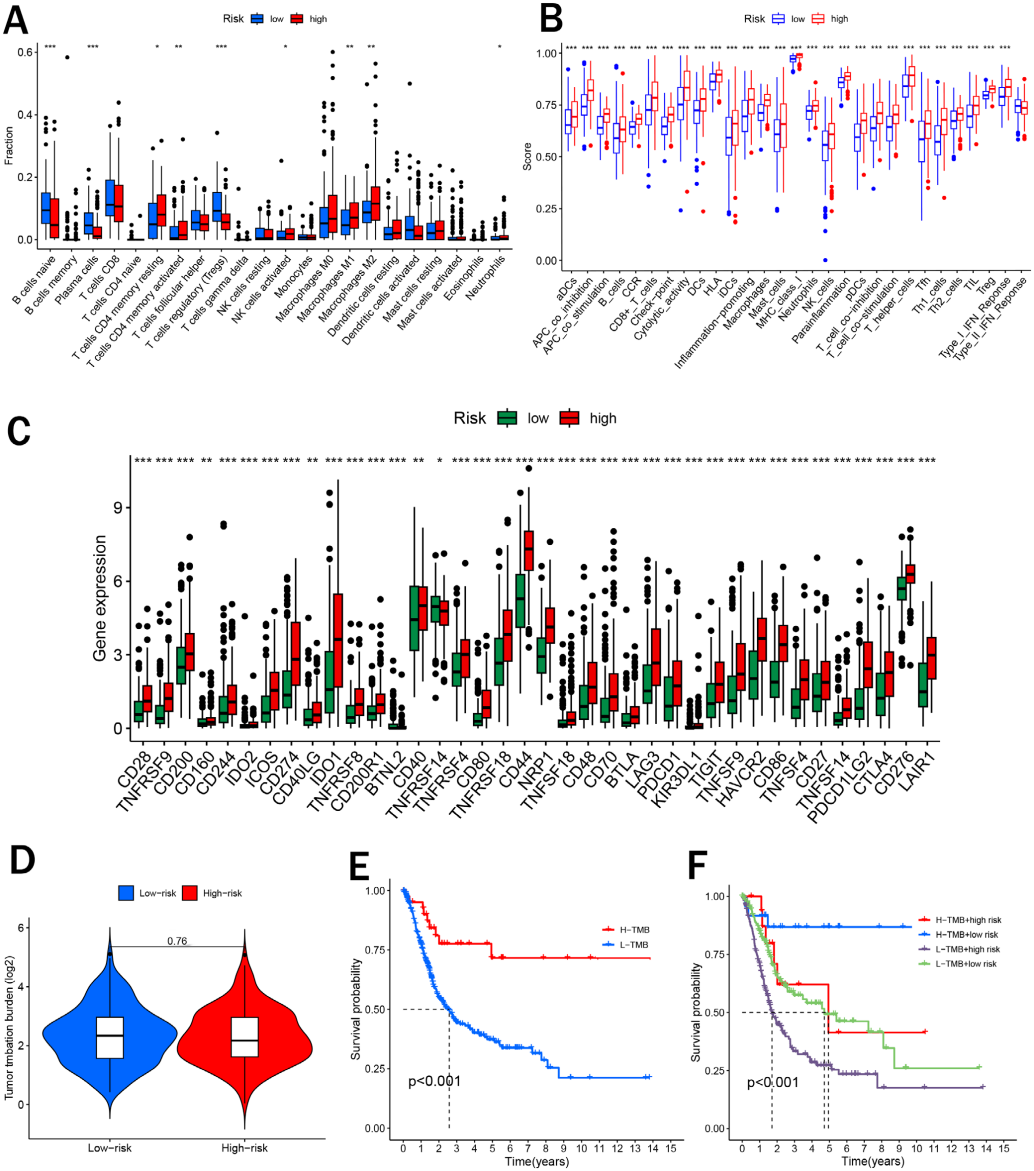
Tumor immune microenvironment analysis. (A) Immunocyte infiltration fractions in two risk groups; (B) ssGSEA scores of 16 immune cells and 13 immune functions in two risk groups; (C) The expression levels of various immune checkpoints in two risk groups; (D) Tumor mutation burden (TMB) analysis in two risk groups; (E) Kaplan–Meier survival analysis for TMB; (F) Kaplan–Meier survival analysis for TMB and risk score. *p < 0.05, **p < 0.01, ***p < 0.001.

Based on the risk signature, we investigated the immune microenvironment in BCa patients. The fractions of B cells naive, plasma cells, and T cells regulatory (Tregs) were significantly higher in the low‐risk group while the fractions of T cells CD4 memory resting, T cells CD4 memory activated, NK cells activated, macrophages M1, and macrophages M2 were higher in the high‐risk group (Figure 5A). Meanwhile, we found that various immune functions, including APC‐co‐inhibition, checkpoint, cytolytic activity, inflammation promoting, MHC‐class‐I, parainflammation, T cell co‐inhibition, and Type I IFN Response all scored higher in the high‐risk group (Figure 5B). These findings elucidated that immune functions may be more active in high‐risk BCa patients and these patients may exhibit better efficacy in immunotherapy. We also examined several immune checkpoints in two risk groups. The results revealed that a majority of immune checkpoints such as CD28, TNFRSF9, CD200, IDO1, CTLA4, and so on, possessed higher expression in high‐risk BCa patients (Figure 5C). Thereby, we could infer that high‐risk BCa patients may be more sensitive to potential immune checkpoint therapy. Besides, we compared the tumor mutation burden (TMB) and found that TMB showed no statistically significant difference in two risk groups (Figure 5D). Survival analysis pointed out that BCa patients with a higher TMB exhibited better prognosis (Figure 5E). Furthermore, we found that patients with the lowest risk score and highest TMB showed the best survival status whereas those with the highest risk score and lowest TMB showed the poorest survival status (Figure 5F).

### 3.6 Cluster Analysis Based on the Risk Signature

**FIGURE 6:**
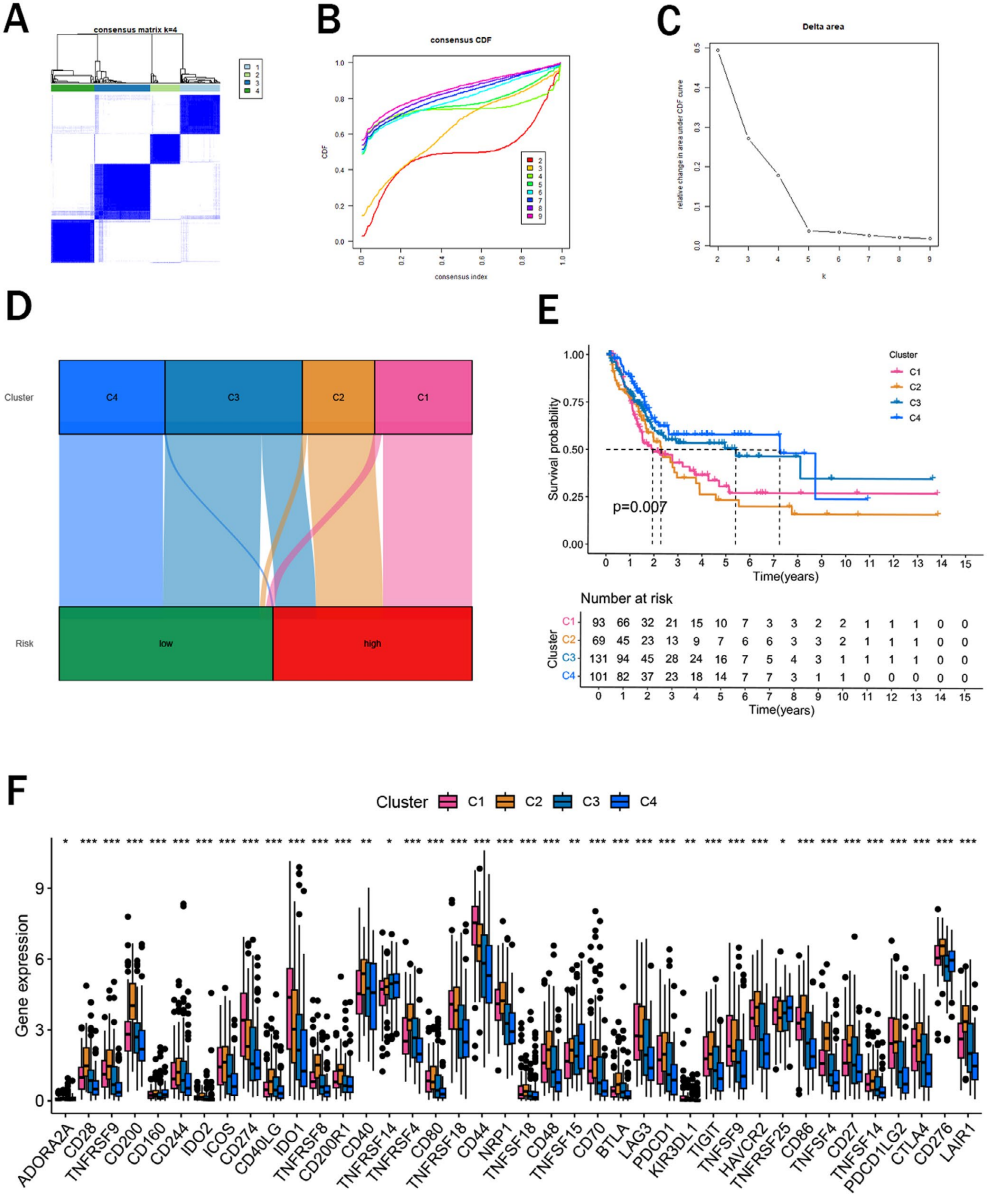
Cluster analysis. (A) All patients were categorized into two clusters according to the consensus clustering matrix (*k* = 4); (B) Area under the cumulative distribution function (CDF) curve when *k* = 2–9; (C) Changes in the length and inclination of the CDF curve when *k* = 2–9; (D) Sankey diagram of the four clusters and two risk groups; (E) Kaplan–Meier survival analysis for the four clusters; (F) The expression levels of immune checkpoints in the four clusters. *p < 0.05, **p < 0.01, ***p < 0.001.

To further explore the correlation between risk signature and different tumor subtypes, we conducted a cluster analysis and categorized all BCa patients into four clusters (Figure 6A–C). As shown in Figure 6D, most high‐risk patients belonged to cluster 1 and 2, while most low‐risk patients were separated into cluster 3 and 4. As expected, survival analysis indicated that cluster 3 and 4 had an obviously higher survival rate than cluster 1and 2 (Figure 6E). What's more, various immune checkpoints were all more expressed in cluster 1 and 2, consistent with previous results (Figure 6F).

### 3.7 Drug Sensitivity Analysis Based on the Risk Signature

**FIGURE 7:**
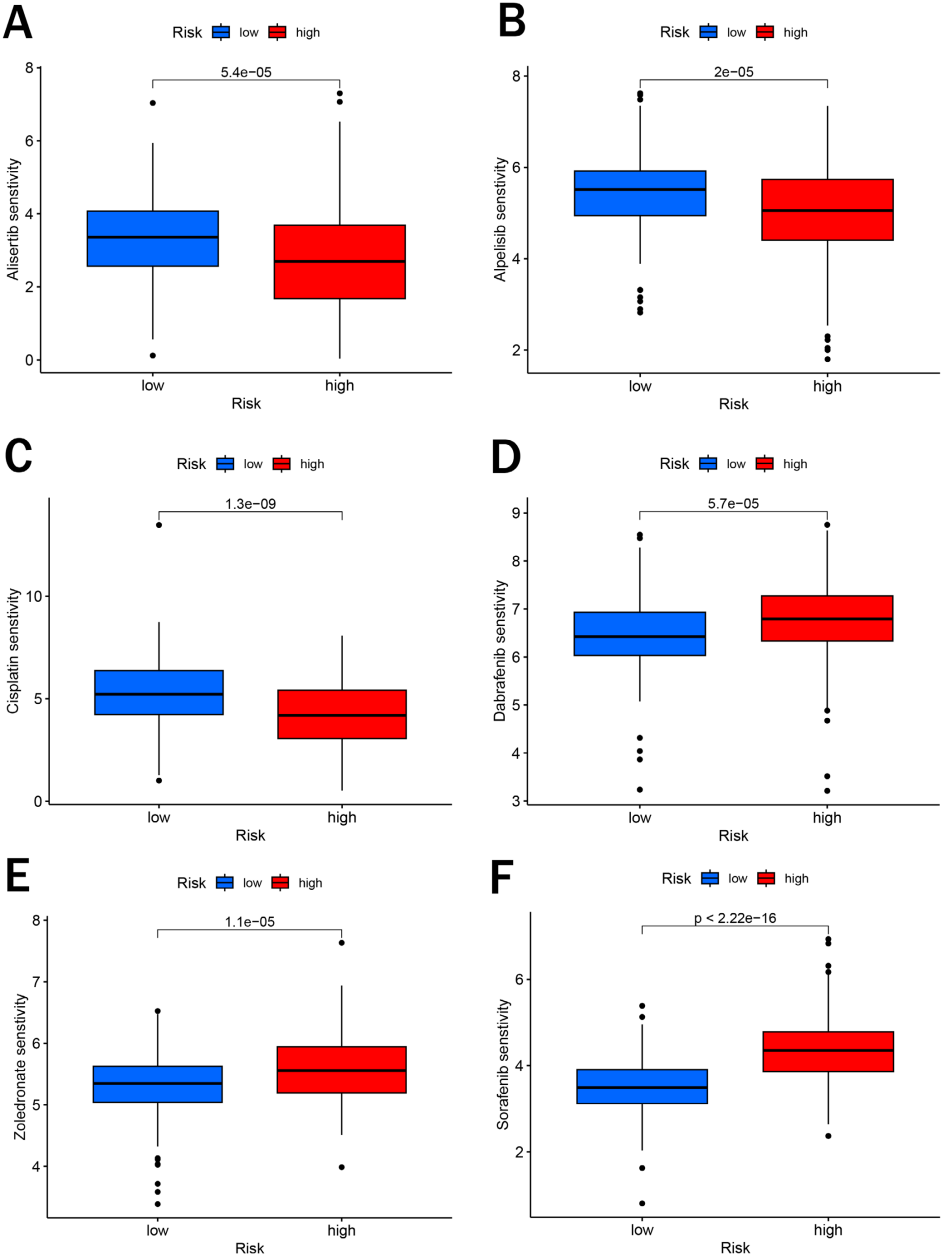
Drug sensitivity analysis. (A–F) IC50 of several chemotherapy drugs in BCa. (A) Alisertib; (B) Alpelisib; (C) Cisplatin; (D) Dabrafenib; (E) Zoledronate; (F) Sorafenib.

To explore the relationship between risk scores and drug sensitivity in BCa patients, we used IC50 values to quantify the sensitivity of various chemical and targeted drugs. Patients in the high‐risk group may be more sensitive to drugs, such as alisertib, alpelisib, and Cisplatin (Figure 7A–C). Conversely, the IC50 values of several drugs, including dabrafenib, zoledronate, and sorafenib, were much higher in the high‐risk group, indicating that these drugs would exhibit better therapeutic effects in low‐risk BCa patients (Figure 7D–F). Our results revealed that the risk signature could serve as a predictor of the therapeutic effects of chemical and targeted drugs for BCa patients.

### 3.8 Drug Enrichment and Molecular Docking

**FIGURE 8:**
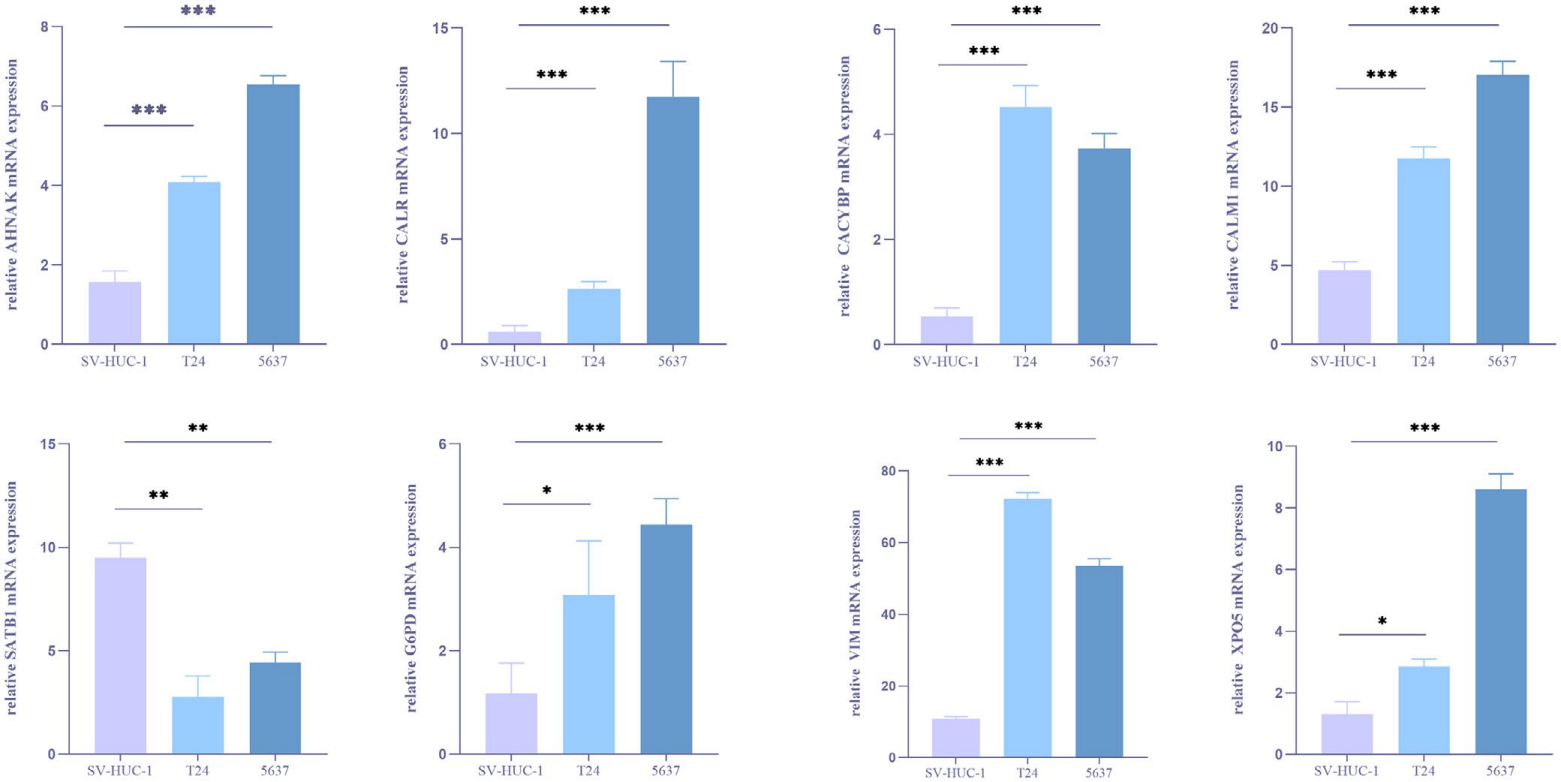
Expression of the lactylation‐related genes in bladder cancer cell lines. *p < 0.05; **p < 0.01; ***p < 0.001.

We performed drug enrichment analysis to identify potential therapeutic compounds associated with the 25 prognostic lactylation‐related genes. All potential therapeutic drugs associated with the prognostic genes, ranked by enrichment significance, are listed in Table 2. Figure 8A illustrated the relationships between the top ten enriched drugs and their associated genes. We generated a bubble plot, arranging the enriched drugs according to their adjusted *p*‐values and the corresponding number of associated genes (Figure 8B), with AG‐012559 being the drug exhibiting the highest level of enrichment. However, AG‐012559 is an experimental drug that is either still in the experimental phase or has been discontinued, making it unavailable for 3D structure retrieval. Therefore, we selected the drug with the second‐highest enrichment significance for subsequent molecular docking analysis. The docking modes of the potential therapeutic drug “Carcinine” with its three associated genes (CALR, ENO1, HMGA1) were displayed in the Figure 8C–E.

**TABLE 2:** Potential drug enrichment results for prognostic genes.

ID	Description	*p*	*p*.adjust	*q*	geneID	Count
AG‐012559	AG‐012559	8.87E‐07	0.000406	0.000216	ENO1/GAPDH/PPIA/PRKDC/UBE2M	5
Carcinine	Carcinine	1.53E‐06	0.000406	0.000216	CALR/ENO1/HMGA1	3
Oxetacaine	Oxetacaine	4.09E‐06	0.000722	0.000384	CALR/ENO1/HMGA1	3
Salsolidin	Salsolidin	5.73E‐06	0.000759	0.000404	CALR/ENO1/HMGA1	3
Gly‐His‐Lys	Gly‐His‐Lys	1.02E‐05	0.000859	0.000457	ENO1/GAPDH/PRKDC	3
Cefalonium	Cefalonium	1.11E‐05	0.000859	0.000457	CALR/ENO1/HMGA1	3
Diltiazem	Diltiazem	1.14E‐05	0.000859	0.000457	CALM1/CALR/ENO1/GAPDH/HMGA1/PRKDC	6
Cefotiam	Cefotiam	1.60E‐05	0.000859	0.000457	CALR/ENO1/HMGA1/PRKDC	4
2‐Bromo‐3‐hydroxy‐4‐methoxybenzaldehyde	2‐Bromo‐3‐hydroxy‐4‐methoxybenzaldehyde	1.65E‐05	0.000859	0.000457	CACYBP/GAPDH/HMGA1/PPIA	4
2‐Nonenal, 4‐hydroxy‐, (2E,4R)—	2‐Nonenal, 4‐hydroxy‐, (2E,4R)—	1.74E‐05	0.000859	0.000457	ENO1/HMGA1/PRKDC/SATB1/VIM	5
Amprolium	Amprolium	1.78E‐05	0.000859	0.000457	CALR/ENO1/HMGA1	3
PHA‐00665752	PHA‐00665752	2.21E‐05	0.000977	0.00052	CALM1/ENO1/GAPDH/PRKDC/UBE2M	5
STOCK1N‐28457	STOCK1N‐28457	2.85E‐05	0.00116	0.000617	ENO1/GAPDH/PRKDC/UBE2M	4
Meglumine	Meglumine	3.21E‐05	0.001215	0.000647	CALR/ENO1/HMGA1	3
PNU‐0293363	PNU‐0293363	3.66E‐05	0.001294	0.000689	CALM1/ENO1/GAPDH/GATAD2A/PRKDC	5
H‐89	H‐89	6.97E‐05	0.002308	0.001229	ENO1/GAPDH/UBE2M	3
Ibuprofen	Ibuprofen	7.58E‐05	0.002364	0.001258	ENO1/PABPN1/PPIA/RAN	4
Adenosine triphosphate	Adenosine triphosphate	9.08E‐05	0.002673	0.001423	ENO1/GAPDH/PFKM/VIM	4
Doxylamine	Doxylamine	0.000129	0.003528	0.001878	CALR/HMGA1	2
PHA‐00767505E	PHA‐00767505E	0.000133	0.003528	0.001878	ENO1/GAPDH/PRKDC	3
[6‐[6‐(butanoylamino)purin‐9‐yl]‐2‐hydroxy‐2‐oxo‐4a,6,7,7a‐tetrahydro‐4H‐furo[3,2‐d][1,3,2]dioxaphosphinin‐7‐yl] butanoate	[6‐[6‐(butanoylamino)purin‐9‐yl]‐2‐hydroxy‐2‐oxo‐4a,6,7,7a‐tetrahydro‐4H‐furo[3,2‐d][1,3,2]dioxaphosphinin‐7‐yl] butanoate	0.000141	0.003563	0.001896	CALM1/PPIA/RAN/VIM	4
7,8‐Benzoflavone	7,8‐Benzoflavone	0.000187	0.004512	0.002401	ENO1/PFKM/PRKDC	3
Succinylsulfathiazole	Succinylsulfathiazole	0.0002	0.004599	0.002448	CALR/ENO1/HMGA1	3
Corynanthine	Corynanthine	0.000216	0.004775	0.002541	CALR/HMGA1	2
KU‐55933	KU‐55933	0.000241	0.005119	0.002725	PRKDC/TRIM28	2
PF‐00562151‐00	PF‐00562151‐00	0.000296	0.006036	0.003213	ENO1/GAPDH	2
Orciprenaline	Orciprenaline	0.00042	0.007983	0.004249	CALR/ENO1/HMGA1	3
FERRIC AMMONIUM CITRATE	FERRIC AMMONIUM CITRATE	0.000422	0.007983	0.004249	GAPDH/PPIA	2
Benfotiamine	Benfotiamine	0.000495	0.009039	0.004811	CALM1/CALR/HMGA1	3
SC‐560	SC‐560	0.00053	0.009367	0.004986	ENO1/GAPDH	2
AH‐23848	AH‐23848	0.000553	0.009425	0.005017	ENO1/GAPDH/PPIA	3
SR‐95531	SR‐95531	0.000569	0.009425	0.005017	CALR/ENO1	2
Okadaic acid	Okadaic acid	0.000629	0.010103	0.005377	CACYBP/GAPDH/PPIA	3
Cobalt sulfate	Cobalt sulfate	0.000694	0.010812	0.005755	ENO1/GAPDH	2
7‐aminocephalosporanic acid	7‐aminocephalosporanic acid	0.00083	0.012039	0.006408	CALR/ENO1	2
Fenbuconazole	Fenbuconazole	0.00083	0.012039	0.006408	CALR/GAPDH	2
Amikacin	Amikacin	0.000845	0.012039	0.006408	ENO1/GAPDH/PRKDC/VIM	4
Simvastatin	Simvastatin	0.000863	0.012039	0.006408	CALD1/CALR/ENO1/HMGA1	4
16,16‐dimethylprostaglandin E2	16,16‐dimethylprostaglandin E2	0.001031	0.013575	0.007226	ENO1/GAPDH	2
AC1L1I3Y	AC1L1I3Y	0.001031	0.013575	0.007226	G6PD/GAPDH	2
GLYCOGEN	GLYCOGEN	0.001071	0.013575	0.007226	ENO1/PFKM/VIM	3
Bandrowski's base	Bandrowski's base	0.001084	0.013575	0.007226	G6PD/PPIA	2
ACRYLAMIDE	ACRYLAMIDE	0.001127	0.013575	0.007226	ENO1/GAPDH/VIM	3
Hyoscyamine	Hyoscyamine	0.001127	0.013575	0.007226	ENO1/TRIM28/UBE2M	3
5‐azacytidine	5‐azacytidine	0.001175	0.013758	0.007323	CALD1/GAPDH/HMGA1/VIM	4
Phosphoenolpyruvate	Phosphoenolpyruvate	0.001195	0.013758	0.007323	ENO1/PFKM	2
Medroxyprogesterone acetate	Medroxyprogesterone acetate	0.00122	0.013758	0.007323	CALM1/PPIA/RAN/VIM	4
Benserazide	Benserazide	0.001328	0.014534	0.007736	ENO1/HMGA1/PRKDC	3
Indoprofen	Indoprofen	0.001371	0.014534	0.007736	CALR/ENO1	2
Toluidine Blue O	Toluidine Blue O	0.001371	0.014534	0.007736	ENO1/VIM	2
CHROMIUM	CHROMIUM	0.001437	0.014935	0.00795	CALR/HMGA1/PRKDC	3
Amantadine	Amantadine	0.001528	0.015574	0.00829	CALR/ENO1/HMGA1	3
Isoguanine	Isoguanine	0.001599	0.015985	0.008509	ENO1/PPIA/VIM	3
Piperine	Piperine	0.001897	0.01862	0.009911	CALR/ENO1	2
Deferoxamine	Deferoxamine	0.002009	0.019196	0.010218	ENO1/GAPDH/PPIA	3
CP‐863187	CP‐863187	0.002041	0.019196	0.010218	ENO1/PRKDC	2
Lactic acid	Lactic acid	0.002064	0.019196	0.010218	ENO1/G6PD/PFKM	3
3‐Butylidenephthalide	3‐Butylidenephthalide	0.002267	0.020718	0.011028	CALR/ENO1	2
Vorinostat	Vorinostat	0.002331	0.020814	0.011079	CALR/ENO1/PPIA/PRKDC	4
Buflomedil	Buflomedil	0.002356	0.020814	0.011079	CALR/ENO1/HMGA1	3
Iniprol	Iniprol	0.002586	0.021935	0.011675	ENO1/VIM	2
Acetaldehyde	Acetaldehyde	0.002607	0.021935	0.011675	GAPDH/PSMA7/VIM	3
Dexibuprofen	Dexibuprofen	0.002607	0.021935	0.011675	ENO1/GAPDH/PRKDC	3
Mustard gas	Mustard gas	0.002739	0.022681	0.012073	AHNAK/CALM1/GAPDH	3
Epinephrine	Epinephrine	0.00284	0.023158	0.012327	ENO1/GAPDH/VIM	3
3‐(1‐methylpyrrolidin‐2‐yl)pyridine	3‐(1‐methylpyrrolidin‐2‐yl)pyridine	0.002918	0.023429	0.01247	CALR/HMGA1/TRIM28/VIM	4
UNII‐9XX54M675G	UNII‐9XX54M675G	0.003012	0.023829	0.012684	CALD1/CSRP1	2
Uranium acetate	Uranium acetate	0.003122	0.024329	0.01295	PPIA/RAN/TRIM28	3
Ifosfamide	Ifosfamide	0.003192	0.024466	0.013022	ENO1/VIM	2
URANIUM	URANIUM	0.003231	0.024466	0.013022	PPIA/RAN/TRIM28	3
HEMATOXYLIN	HEMATOXYLIN	0.003381	0.025241	0.013435	ENO1/GAPDH/VIM	3
Topotecan	Topotecan	0.003497	0.025739	0.0137	HMGA1/SATB1/VIM	3
Belinostat	Belinostat	0.003565	0.02588	0.013775	RAN/VIM	2
Pyrantel	Pyrantel	0.003661	0.02622	0.013956	ENO1/TRIM28	2
Dorzolamide	Dorzolamide	0.003816	0.026898	0.014317	CALR/ENO1/PRKDC	3
Orlistat	Orlistat	0.003857	0.026898	0.014317	ENO1/GAPDH	2
Chlorpromazine	Chlorpromazine	0.003982	0.027057	0.014402	CALM1/G6PD/PPP1CB	3
Ellagic acid	Ellagic acid	0.003982	0.027057	0.014402	CALR/GAPDH/RAN	3
Dibenzo[def,p]chrysene	Dibenzo[def,p]chrysene	0.00469	0.031464	0.016747	ENO1/G6PD	2
HEXANE	HEXANE	0.00491	0.032457	0.017276	G6PD/VIM	2
Zinc sulfate	Zinc sulfate	0.004975	0.032457	0.017276	G6PD/PABPN1/PFKM	3
Oxolinic acid	Oxolinic acid	0.005022	0.032457	0.017276	TRIM28/UBE2M	2
DMBA	DMBA	0.005249	0.033516	0.01784	GAPDH/PFKM	2
Diallyl trisulfide	Diallyl trisulfide	0.005598	0.035322	0.018801	ENO1/GAPDH	2
Nitrofural	Nitrofural	0.00573	0.035728	0.019017	ENO1/GAPDH/PRKDC	3
Methylene blue	Methylene blue	0.006454	0.039774	0.021171	G6PD/VIM	2
0297417‐0002B	0297417‐0002B	0.006551	0.039909	0.021243	CALD1/ENO1/PRKDC	3
BAS‐012416453	BAS‐012416453	0.006838	0.041182	0.02192	ENO1/PRKDC	2
Pipemidic acid	Pipemidic acid	0.007365	0.043862	0.023346	ENO1/PRKDC	2
4‐PHENYLBUTYRIC ACID	4‐PHENYLBUTYRIC ACID	0.007636	0.044968	0.023935	HMGA1/XPO5	2
R‐atenolol	R‐atenolol	0.007814	0.045509	0.024223	CALR/ENO1/HMGA1	3
Pentetrazol	Pentetrazol	0.00805	0.046376	0.024685	ENO1/TRIM28	2
Fulvestrant	Fulvestrant	0.008596	0.048591	0.025864	CALD1/ENO1/GAPDH	3
1,10‐phenanthroline	1,10‐phenanthroline	0.008618	0.048591	0.025864	ENO1/GAPDH	2
Yohimbic acid	Yohimbic acid	0.008763	0.048886	0.026021	ENO1/PABPN1	2
Methylergometrine	Methylergometrine	0.008867	0.048951	0.026055	PFKM/PRKDC/SATB1	3

### 3.9 Expression Levels of the Model Genes in BCa

**FIGURE 9:**
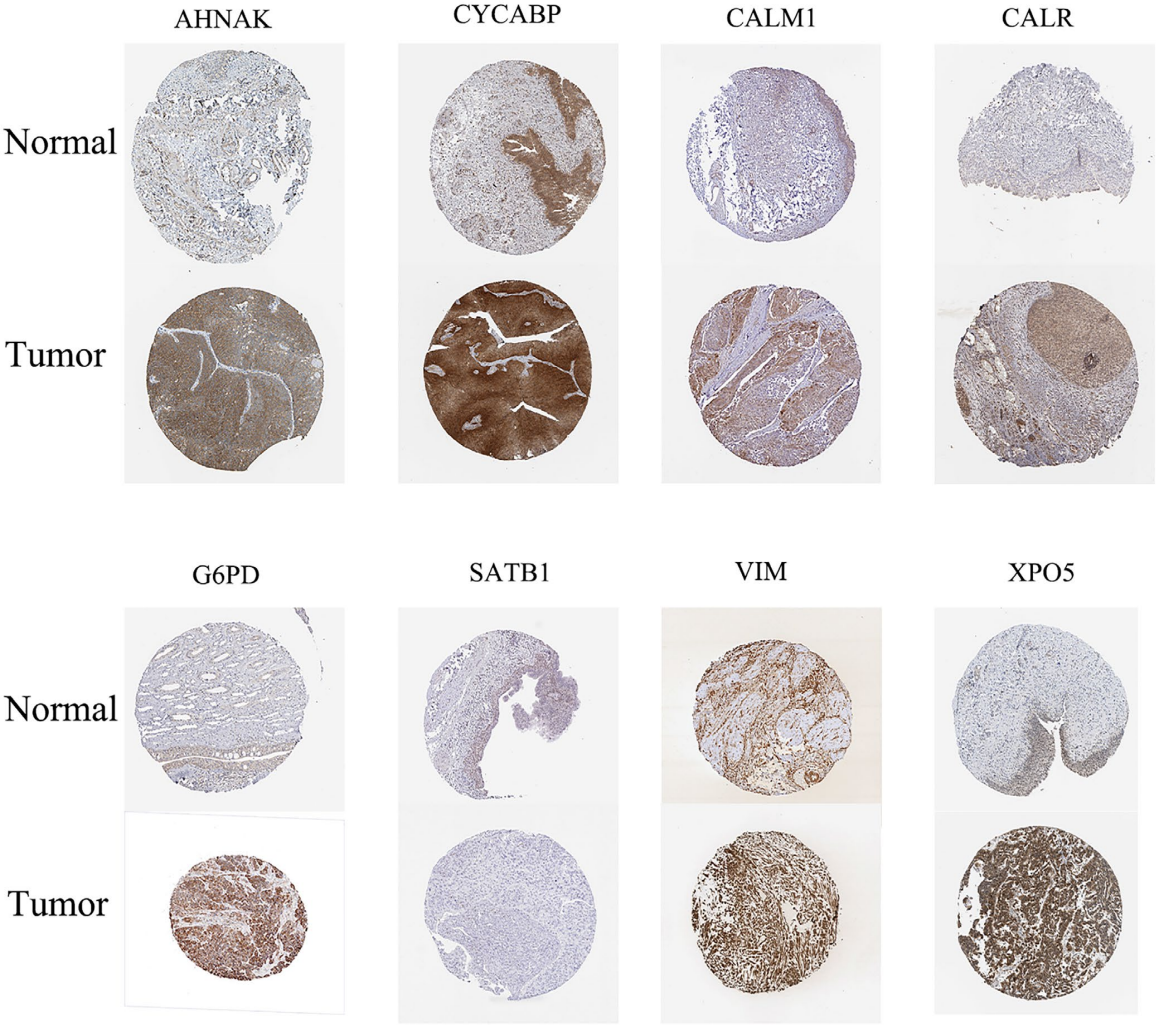
Protein expression levels of model genes in bladder cancer and adjacent tissues from the Human Protein Atlas database.

**FIGURE 10:**
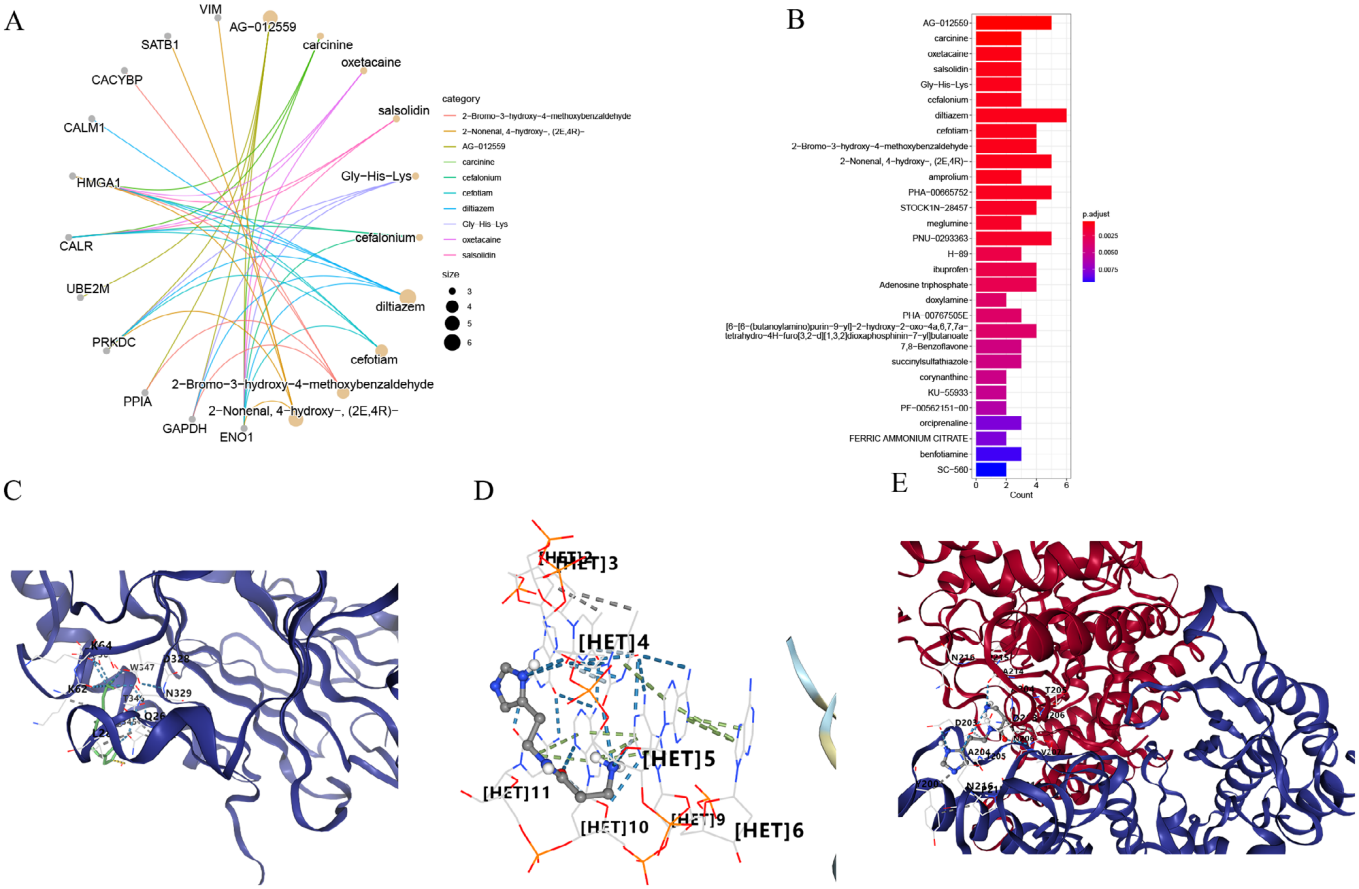
Drug enrichment analysis of prognostic genes and molecular docking. (A and B) 25 prognostic genes and significantly enriched potential therapeutic drugs; (C–E) The docking modes of drug “Carcinine” with its three associated genes (CALR, ENO1, HMGA1).

The RT‐PCR was performed to detect the expression level of the lactylation‐related genes in the risk signature. As is displayed in Figure 9, AHNAK, CALM1, CALR, G6PD, CALM1, CACYBP and VIM were all more highly expressed in two BCa cell lines than normal urinary epithelial cells while SATB1 showed a contrary tendency. By querying the Human Protein Atlas, we obtained the immunohistochemical results of the model genes in bladder cancer. Exactly as revealed in Figure 10, the immunohistochemical results were consistent with our PCR outcomes.

### 3.10 The Knockdown of AHNAK Inhibit Bladder Cancer Cells Proliferation, Migration, and Invasion, While Promoting Apoptosis

**FIGURE 11:**
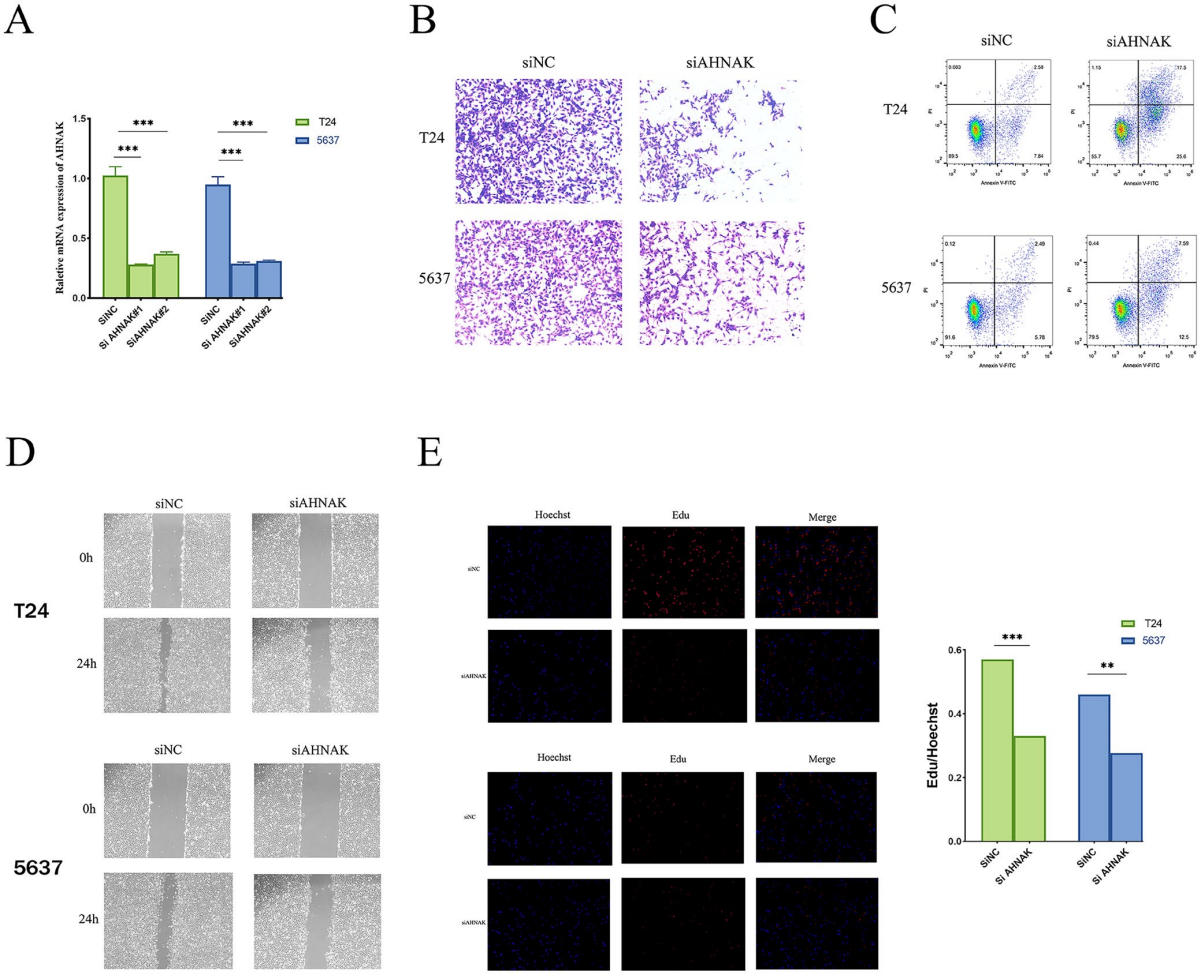
The knockdown of AHNAK inhibits bladder cancer cells proliferation, migration, and invasion, while promoting apoptosis. (A) RT‐qPCR analysis verified the successful knockdown of ISOC1 in both T24 and 5637 cells; (B) Knockdown of AHNAK inhibits the invasion of bladder cancer cells; (C) Knockdown of AHNAK promotes apoptosis in bladder cancer cells; (D) Knockdown of AHNAK reduces the migratory capacity of bladder cancer cells; (E) Knockdown of AHNAK inhibits the proliferative capacity of bladder cancer cells. **p < 0.01; ***p < 0.001.

To further validate the impact of our prognostic model on bladder cancer, a series of in vitro experiments were performed. Previous analyses revealed that AHNAK exhibited the strongest correlation with bladder cancer among the prognostic genes, prompting its selection for subsequent investigations. Two siRNAs were designed to target and knock down AHNAK. RT‐qPCR results confirmed high knockdown efficiency for both siRNAs (Figure 11A), and the one with the superior knockdown efficacy was selected for further studies. The EdU (Figure 11E), Transwell Invasion (Figure 11B), and wound healing assays (Figure 11D) collectively demonstrated that AHNAK knockdown significantly suppressed bladder cancer cell proliferation, migration, and invasion. Moreover, apoptosis assays revealed that the depletion of AHNAK enhanced apoptosis in bladder cancer cells (Figure 11C).

## 4 Discussion

Bladder cancer is a heterogeneous disease with varying clinical outcomes, highlighting the need for reliable prognostic markers and effective treatment strategies. In this study, we aimed to identify a lactylation‐related gene signature that could serve as a predictive tool for prognosis and response to immunotherapy in BCa patients.

Lactylation, a newly discovered post‐translational modification, involves the attachment of lactate molecules to lysine residues on proteins [12]. This process has garnered significant attention in recent years due to its potential implications in various cellular functions and disease states, including cancer [24]. Lactylation plays a multifaceted role in cancer biology, influencing key cellular processes such as metabolism, signaling pathways, and immune responses. In the context of cancer, dysregulation of lactylation processes can contribute to tumor development, progression, and therapeutic responses [25, 26, 27]. One of the primary effects of lactylation in cancer is its impact on cellular metabolism. Lactylation can lead to metabolic rewiring, promoting glycolysis and lactate production even in the presence of oxygen, a phenomenon known as the Warburg effect. This metabolic shift not only provides energy for rapidly proliferating cancer cells but also creates a unique tumor microenvironment characterized by acidic pH and immunosuppressive features [28]. Furthermore, lactylation has been implicated in modulating immune responses within the tumor microenvironment. High lactate levels resulting from increased lactylation can inhibit immune cell functions, such as T cell activation and cytokine production, leading to immune evasion by cancer cells. Additionally, lactylation‐related changes in protein structures may affect antigen presentation and immune recognition, influencing the efficacy of immunotherapy interventions [29]. The dysregulation of lactylation processes in cancer presents promising opportunities for therapeutic interventions and biomarker development. Targeting lactylation‐related enzymes or pathways could potentially disrupt cancer cell metabolism and restore immune surveillance, enhancing the effectiveness of existing therapies such as immunotherapy [30].

Our analysis identified 25 lactylation‐related genes significantly associated with the prognosis of BCa patients and finally chose six genes to construct the risk model. Among the identified genes, AHNAK showed the highest frequency of somatic mutations, suggesting its potential as a key driver gene in BCa development. A recent study reported that NAT10 could stabilize AHNAK mRNA by protecting it from the degradation by RNase and the DNA damage repair mediated by AHNAK is the mechanism through which NAT10 induces resistance to cisplatin [31]. This study provided a potential target for overcoming cisplatin resistance in BCa. However, there is a lack of further research and deeper investigation into its specific role and mechanism in BCa. Other genes in our prognostic risk signature have been reported to be closely associated with bladder cancer. For instance, Knocking down CACYBP inhibits the proliferation and migration of bladder cancer cells and promotes apoptosis through the caspase‐3/ELISA pathway, suggested that CACYBP may be an oncogene in bladder cancer [32]. The epigenetic dysregulation of VIM would promote epithelial‐mesenchymal transition, leading to invasion and metastasis of bladder cancer [33]. Chen and partners found that knocking down G6PD promotes the accumulation of reactive oxygen species, leading to cell death in bladder cancer cells, thereby reducing disease progression and improving prognosis [34].

We conducted a drug enrichment analysis on the 25 prognostic genes and found that the most significantly enriched compound potentially effective for bladder cancer treatment is AG‐012559. AG‐012559, also known as AG‐013736 (Axitinib), is an experimental drug primarily classified as an anti‐angiogenic agent. Its mechanism of action involves the inhibition of vascular endothelial growth factor receptors (VEGFRs), which plays a crucial role in tumor angiogenesis—the formation of new blood vessels that supply tumors with nutrients and oxygen. By blocking the VEGFR signaling pathway, AG‐012559 can potentially reduce tumor vascularization, thereby inhibiting tumor growth and metastasis. However, as of now, AG‐012559 has not received widespread regulatory approval and remains in the experimental phase, with some reports indicating that its development may have been halted. While Axitinib is primarily used for the treatment of advanced renal cell carcinoma (RCC), particularly in cases where other therapies have failed [35, 36]. However, an increasing number of studies have found that it also has significant therapeutic effects on other tumors, such as breast cancer, melanoma, and gastrointestinal tumors [37, 38, 39]. However, there is currently a lack of clinical studies investigating whether it has a beneficial therapeutic effect on bladder cancer. Carcinine, also known as β‐alanylhistamine, is a naturally occurring dipeptide composed of β‐alanine and histamine. It exhibits antioxidant and free radical‐scavenging properties, playing a role in neuroprotection [40]. Carcinine is primarily found in animal tissues and shares similarities with compounds like carnosine, which has been shown to counteract oxidative stress and slow down cellular aging [41]. Research on carcinine has focused on its potential in protecting against oxidative stress and aging‐related damage. Notably, it has been linked to protective effects on vision and eye diseases such as age‐related macular degeneration and glaucoma [42]. Additionally, carcinine has potential applications in the study of neurodegenerative diseases due to its antioxidant properties and ability to mitigate oxidative damage.

Additionally, we performed functional enrichment analysis to gain insights into the biological processes and pathways associated with lactylation‐related genes in BCa. Our results showed enrichment in pathways related to cell cycle regulation, DNA repair mechanisms, and immune response, indicating the multifaceted roles of lactylation‐related genes in BCa progression. Although carcinine possesses antioxidant and anti‐glycation properties, which could theoretically provide indirect benefits in cancer prevention or as an adjunct in therapy, it is not specifically designed or approved for cancer treatment. For cancer patients, standard therapies such as chemotherapy, radiation therapy, targeted therapy, and immunotherapy remain the primary treatment options. While Carcinine's antioxidant and anti‐glycation properties may suggest some potential for research as an adjunctive treatment, there is not yet sufficient clinical evidence to support its efficacy in treating cancer. Future research may explore Carcinine as a supplementary component in cancer therapy, but for now, the established treatments remain the cornerstone of cancer care.

In conclusion, our study provided valuable insights into the role of lactylation‐related genes in BCa prognosis and molecular characteristics. The identified genes and pathways might serve as potential biomarkers for prognosis prediction and therapeutic targeting in BCa. Further experimental validation and functional studies are warranted to elucidate the precise mechanisms underlying lactylation‐related gene dysregulation in BCa progression.

## Author Contributions

**Jingsong Wang:** conceptualization, investigation, validation, methodology, software, formal analysis, data curation, writing – original draft. **Qianxue Lu:** methodology, investigation, validation, formal analysis. **Panpan Jiao:** methodology, software, formal analysis, investigation, data curation. **Jun Jian:** validation, formal analysis, investigation. **Qingyuan Zheng:** investigation, visualization, supervision, data curation, resources, writing – review and editing. **Zhiyuan Chen:** project administration, writing – review and editing, resources. **Xiuheng Liu:** writing – review and editing, project administration, resources. **Shanshan Wan:** project administration, writing – review and editing, validation, resources, formal analysis. **Lei Wang:** writing – review and editing, funding acquisition, project administration, supervision, resources.

## Funding

This study was funded by “National Natural Science Foundation of China, Grant No. (82000639)”.

## Ethics Statement

The authors have nothing to report.

## Consent

The authors have nothing to report.

## Conflicts of Interest

The authors declare no conflicts of interest.

## Supporting information


**Figure S1:** Flowchart of this study.

**Figure S2:** The AUC of prognostic model in train cohort, test cohort, and two GEO validation cohorts.

## Data Availability

The datasets generated and analyzed during the current study are available in the Cancer Genome Atlas Program (TCGA), https://portal.gdc.cancer.gov/ and the Gene Expression Omnibus database (GEO), https://www.ncbi.nlm.nih.gov/geo/. The immunohistochemical results of model genes are sourced from the Human Protein Atlas (https://www.proteinatlas.org/).
