# Supplementary figures and images for: Machine Learning Integration Framework Constructs a Lactylation‐Associated Gene Signature to Improve Prognosis in Bladder Cancer
Source: Cancer Med. 2026 Jan 8;15(1):e71477. doi: 10.1002/cam4.71477 (PMC12782153; doi:10.1002/cam4.71477)

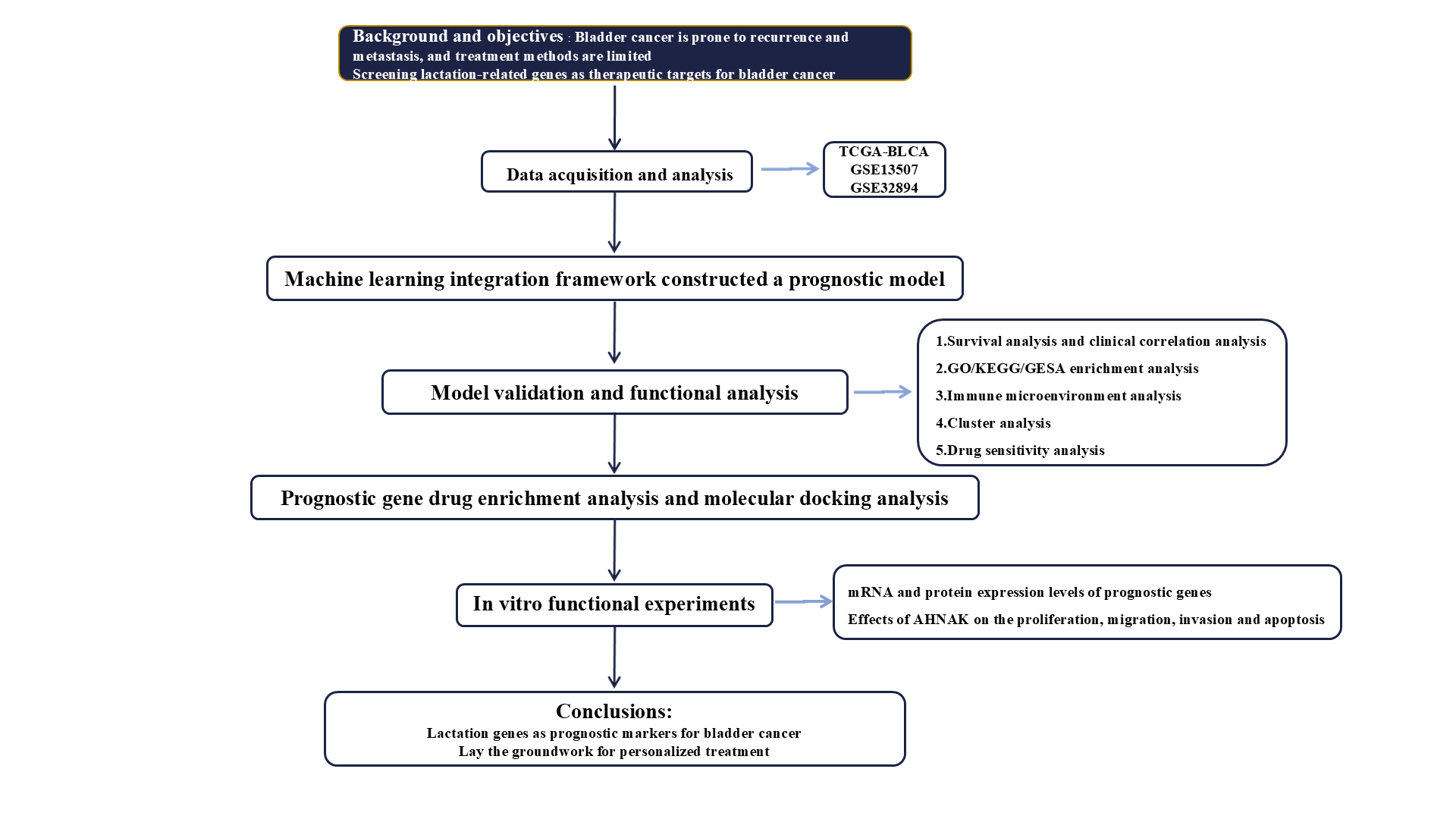

Supplement: Supplementary file 1 — Figure S1: Flowchart of this study. [file CAM4-15-e71477-s001.tiff]

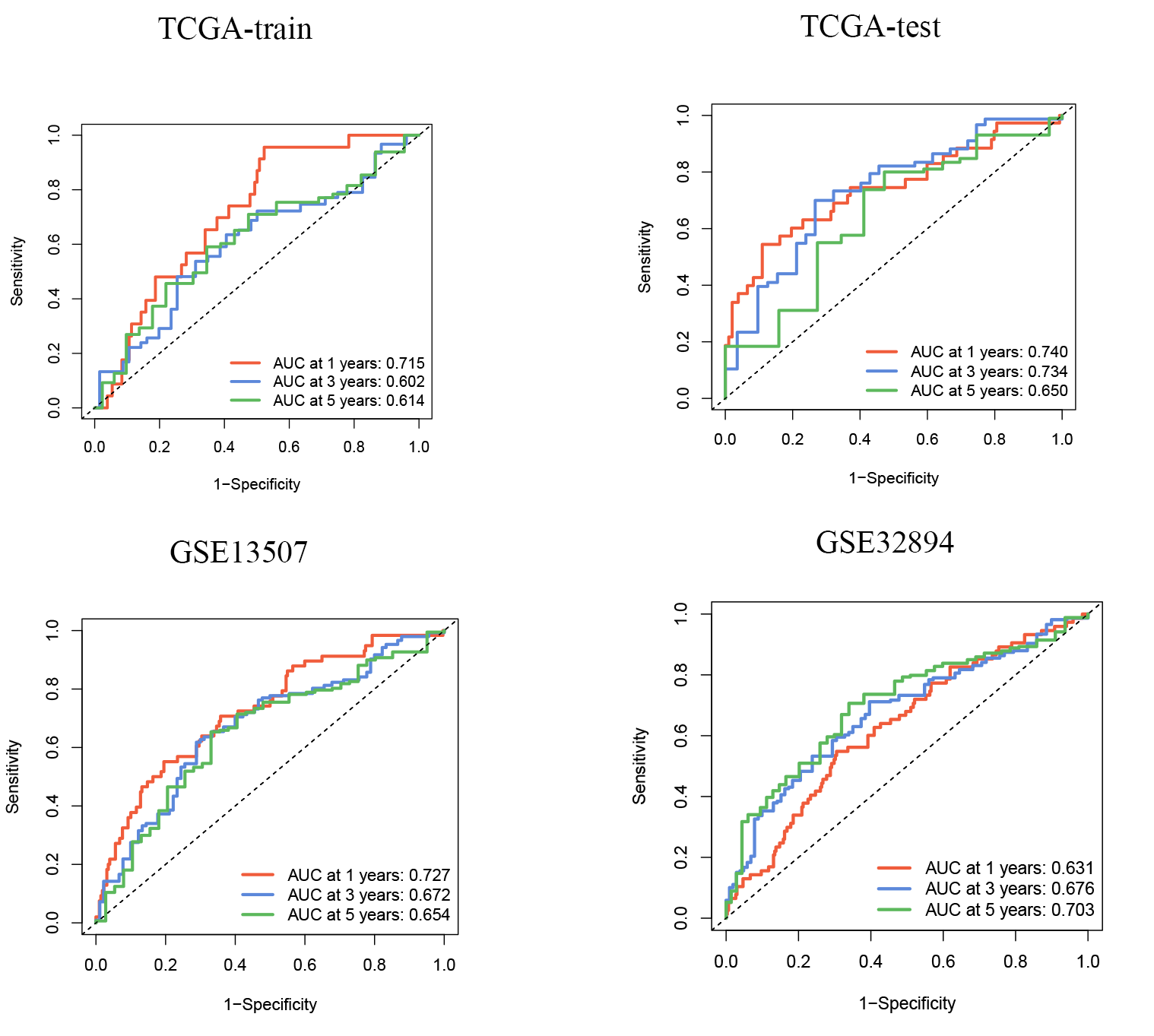

Supplement: Supplementary file 2 — Figure S2: The AUC of prognostic model in train cohort, test cohort, and two GEO validation cohorts. [file CAM4-15-e71477-s002.tif]
